# Identification of B cell marker genes based on single-cell sequencing to establish a prognostic model and identify immune infiltration in osteosarcoma

**DOI:** 10.3389/fimmu.2022.1026701

**Published:** 2022-12-07

**Authors:** Zhongmin Zhang, Jin Zhang, Yuansheng Duan, Xuesong Li, Jie Pan, Guowen Wang, Bin Shen

**Affiliations:** ^1^Department of Bone and Soft Tissue Tumors, Tianjin Medical University Cancer Institute and Hospital, National Clinical Research Center for Cancer, Key Laboratory of Cancer Prevention and Therapy, Tianjin’s Clinical Research Center for Cancer, Tianjin, China; ^2^Department of Maxillofacial and Otorhinolaryngological Oncology, Tianjin Medical University Cancer Institute and Hospital, Key Laboratory of Cancer, Prevention and Therapy, Tianjin Cancer Institute, National Clinical Research Center of Cancer, Tianjin, China; ^3^Department of Pancreatic cancer, Tianjin Medical University Cancer Institute and Hospital, National Clinical Research Center for Cancer, Key Laboratory of Cancer Prevention and Therapy, Tianjin's Clinical Research Center for Cancer, Tianjin, China; ^4^Department of Spine Surgery, Shanghai East Hospital, Tongji University School of Medicine, Shanghai, China

**Keywords:** osteosarcoma, prognosis, immunity, B cell marker gene, single-cell sequencing

## Abstract

**Background:**

Tumor-infiltrating B cells play a crucial role in the promotion or inhibition of tumor development. However, the role of B cells in osteosarcoma remains largely unknown. The aim of this study was to investigate the effect of B cells on the prognosis and immunity infiltration of osteosarcoma.

**Methods:**

Marker genes of B cells were identified based on the single-cell sequencing results of osteosarcoma in the GEO database. The prognostic model was established by the TCGA database and verified by the GEO data. The divergence in immune infiltration between the low-risk and high-risk groups was then compared according to the established prognostic model. Finally, the differential genes in the low-risk and high-risk groups were enriched and analyzed.

**Results:**

A total of 261 B cell marker genes was obtained by single-cell sequencing and a prognostic model of 4 B cell marker genes was established based on TCGA data. The model was found to have a good prediction performance in the TCGA and GEO data. A remarkable difference in immune infiltration between the low-risk and high-risk groups was also observed. The obtained results were verified by enrichment analysis.

**Conclusion:**

In summary, a prognostic model with good predictive performance was established that revealed the indispensable role of B cells in the development of osteosarcoma. This model also provides a predictive index and a novel therapeutic target for immunotherapy for clinical patients.

## Introduction

Osteosarcoma is a primary malignant bone tumor originating from primitive mesenchymal cells (1). Osteosarcoma has a bimodal age pattern, occurring mainly in teenagers and the elderly (2). The most common treatment options for patients with osteosarcoma include surgery, chemotherapy, radiotherapy, immunotherapy, and/or targeted therapy. The 5-year survival rate of patients with non-metastatic osteosarcoma has been stable at 65-70% in the last few decades but for patients with metastatic osteosarcoma, it is only 20-30% (3, 4).

Immune infiltration has a very significant role in the occurrence and development of cancer. B cells, an important part of the tumor immune infiltration system, significantly impacts tumor growth (5, 6). B cells can exert their anti-tumor effect by producing tumor-specific antibodies, enhancing the anti-tumor response of natural killer cells and T cells, secreting interferon-γ, and maintaining the construction and function of tertiary lymphoid structure (TLS). On the contrary, B cells can facilitate the progression of cancer by producing IL-10, inducing tumor angiogenesis and immunosuppression (7, 8). Currently, single-cell sequencing is used to explore immune infiltration and heterogeneity of tumors (9). This investigation not only aims to probe into the effect of B cells on the prognosis and immune infiltration of patients with osteosarcoma but also to develop a prognostic model to provide clinical treatment guidelines.

## Materials and methods

### Data

A total of 6 single-cell sequencing samples of GSE162454 data were downloaded from the Gene expression omnibus (GEO) database and were used to screen B cell marker genes in patients with osteosarcoma. The clinical follow-up information and RNA-seq data of 88 osteosarcoma patients in the Cancer Genome Atlas (TCGA) database were obtained from UCSC Xena. GSE21257 data were obtained from the GEO database as a validation set. All the data used in this study is public, and the original study of these datasets has been ethically approved.

### Obtaining B cell marker genes

Principal component analysis (PCA) and t-distributed stochastic neighbor embedding (t-SNE) algorithms on single-cell sequencing samples were carried out. The expressed cells were clustered using the “Seurat” package in R software. Genes showing adjusted p-value < 0.05 and |log2 (fold change)|> 0.5 were considered to be marker genes. The “SingleR” package and typical cell marker genes were used for annotation.

### Construction risk scoring model based on TCGA data

Using the screened B cell marker genes, a risk-scoring model based on TCGA data were constructed. Screening of B-cell marker genes associated with osteosarcoma prognosis by univariate Cox and multivariate Cox analyses. For univariate analysis, p-value < 0.01 and p-value < 0.05 for multivariate analysis were considered statistically significant. The prognostic genes were further screened by lasso regression analysis, and the risk coefficient (Coefi) of each gene was computed. The risk score was computed based on the expression of B cell marker genes (Expri) and their corresponding lasso regression coefficient (Coefi) (risk score = $\sum_{n}^{i}$ (coefi × expri)). Patients with osteosarcoma were divided into high-risk and low-risk groups using the median risk score. The overall survival rate of the training set and verification set was evaluated and the subject operating characteristic (ROC) curve was used to estimate the prediction accuracy of the risk score model.

### Analysis of immune infiltration between low-risk and high-risk groups

The CIBERSORT algorithm was used to calculate the abundance of 22 kinds of immune cells in the low-risk and high-risk groups. ssGSEA analysis (single sample Gene Set Enrichment Analysis) was also carried out in the low-risk and high-risk groups to compute the content of 28 kinds of immune cells. The stromal score, immune score, estimates score, tumor purity, and differences in gene expression associated with immune checkpoint blockade (ICB) between the low-risk and high-risk groups were then compared. The correlation between the four B cell marker prognostic genes and 28 kinds of immune cells was computed and the relationship between B cells and the prognosis of osteosarcoma was analyzed.

### Nomogram of the prediction model

Univariate Cox and multivariate Cox analyses of gender, age, metastasis, and risk scores for osteosarcoma patients in the TCGA database were performed. The nomogram was established based on the results, and the time ROC curve and the calibration chart of TCGA data and GEO data were used to estimate the prediction performance of the nomogram.

### Enrichment analysis

The differential genes between the high-risk and low-risk groups were screened and the enrichment of these genes was analyzed through Gene Set Enrichment Analyses (GSEA), Gene Ontology (GO) analyses, and Kyoto Encyclopedia of Genes and Genomes (KEGG) analyses. The differential gene screening multiple was set to 1. Pathways associated with activated B cells were identified through GSEA enrichment analysis and the genes in the pathway concerning the prognosis of osteosarcoma were analyzed.

### Immunohistochemical staining

Tissue samples from 3 cases of osteosarcoma without therapy were obtained from the Cancer Hospital of Tianjin Medical University. Informed consent for participation was obtained from all the patients and the study obtained ethical clearance. Osteosarcoma tissue was fixed. The sections were first stored in an oven at 65°C for 3 hours, dewaxed and subjected to antigen repair, and before closed incubation with 5% bovine serum protein (BSA) for one hour at room temperature. Antibodies were selected from CD20 and the sections were finally incubated with biotinylated goat anti-rabbit secondary antibody for another 30 minutes at 37°C and stained.

### Statistical analysis

Correlations between continuous variables were analyzed by Pearson analysis. T-tests were used for statistical analysis between the two groups, and analysis of variance for multi-group tests. In the absence of special instructions, a p-value < 0.05 was considered statistically significant. All the statistical analysis was implemented with R software (R version 4.2.0). (*, P < 0.05, **, P < 0.01, ***, P < 0.001).

## Results

### Training and validation set of osteosarcoma patient data and the flow of this experiment

**Figure 1:**
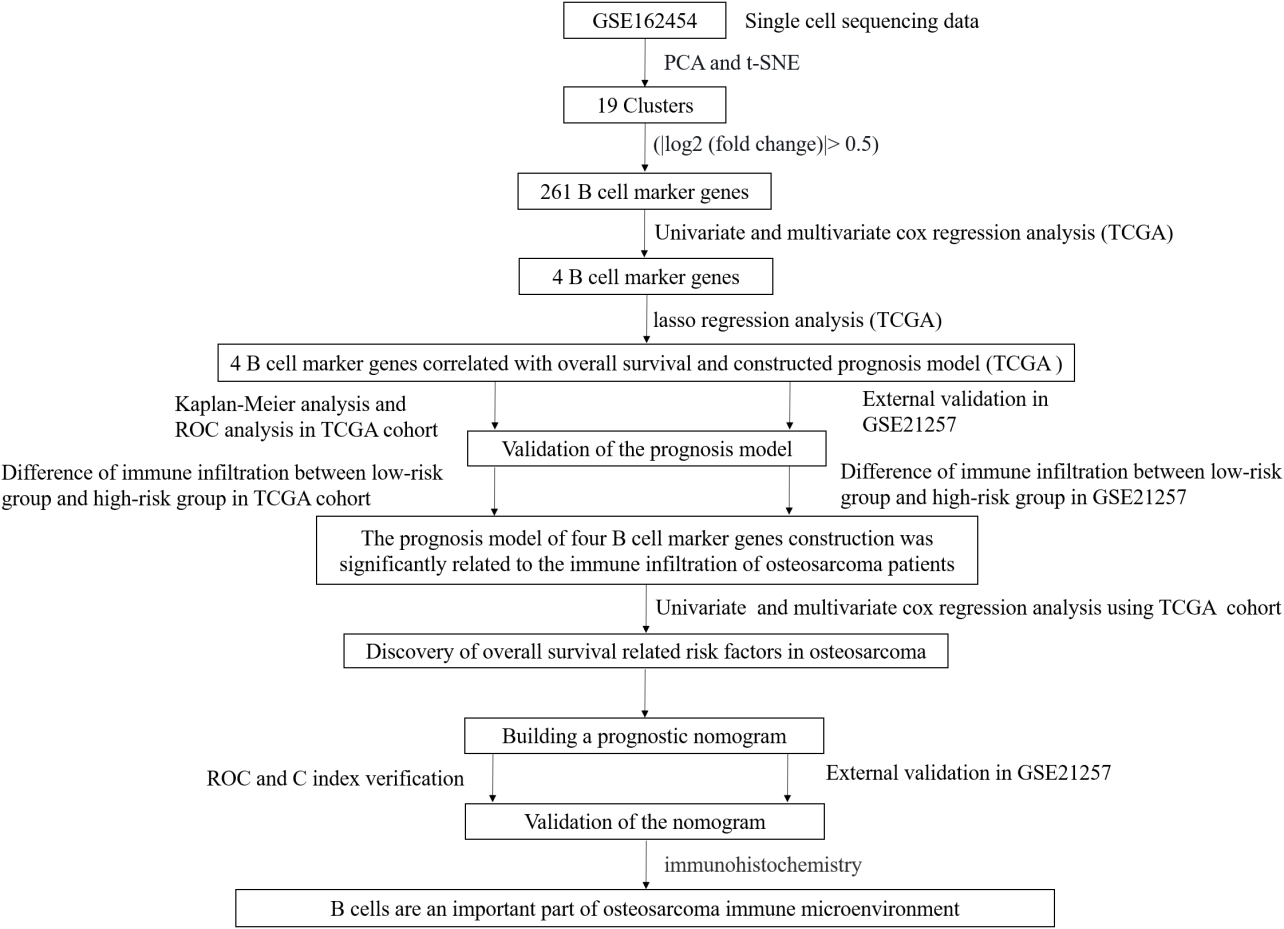
The flow chart describes the research idea and content of this study.

For TCGA data, patients with a lack of survival time and zero survival time were excluded and 84 patients with osteosarcoma were retained. All 53 patients with GSE21257 data were included in this study (**Table S1**). A schematic representation of the study protocol has been shown in **Figure 1**.

### Identification of B cell marker genes based on single-cell sequencing

**Figure 2:**
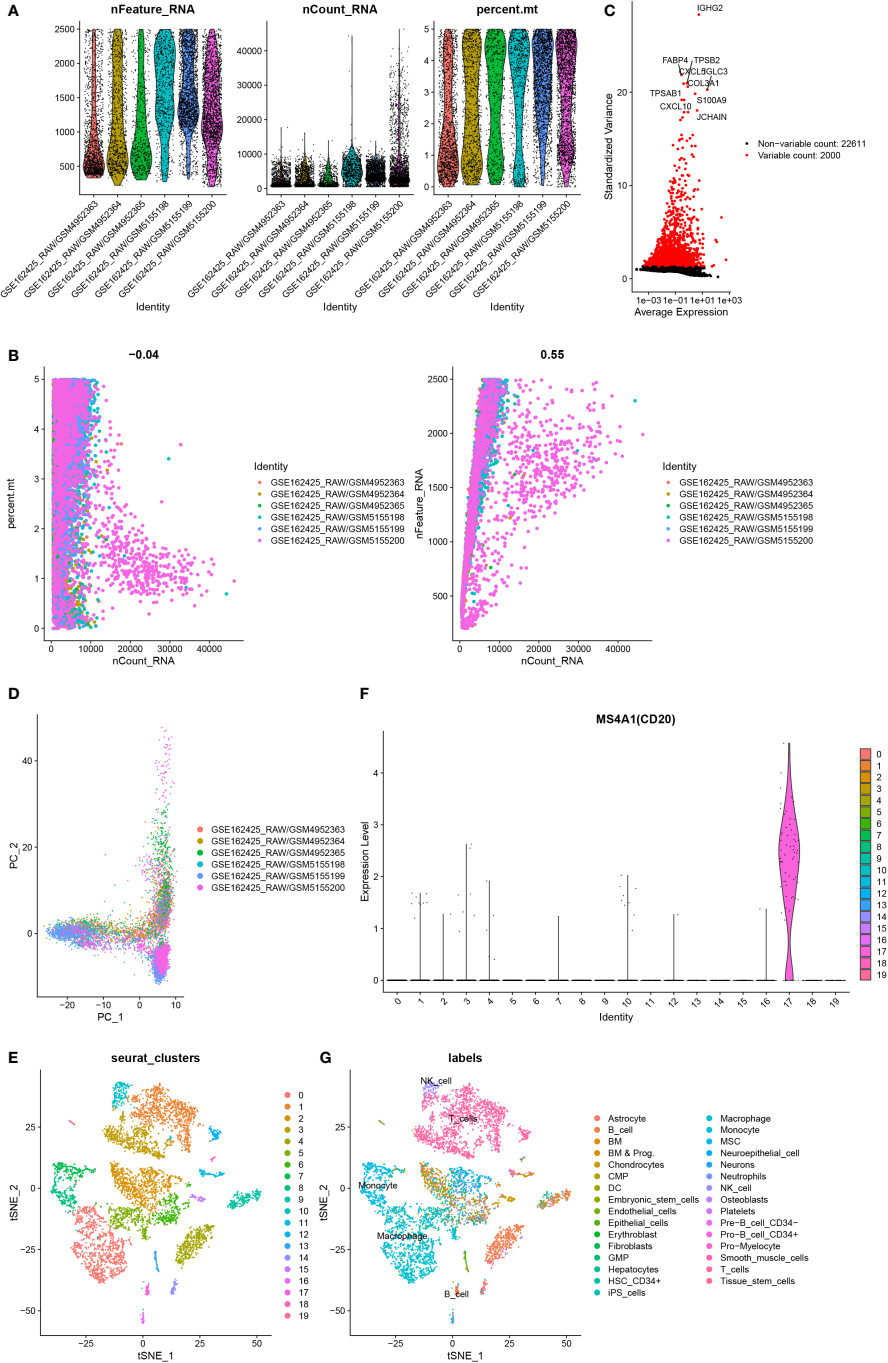
Identification of B cell marker genes by single-cell sequencing samples. **(A)** The violin map shows the gene count of each cell, the sum of all gene expression levels in each cell, and the percentage of mitochondrial genes. **(B)** A scatter plot of the percentage of mitochondrial genes and gene counts in the sum of all gene expression levels in each cell. **(C)** Detection of highly variable genes across the cells. The X-axis represents the average expression, and the y-axis represents standardized variance. **(D)** PCA plot colored by 6 samples. **(E)** t-SNE plot colored by various cell types. **(F)** violin plot of MS4A1 in t-SNE. **(G)** The cell types are identified by marker genes.

Cells having less than 200 genes and more than 5% mitochondrial genes were removed (**Figure 2A, B**). The first 2000 variable expression genes were used for PCA (**Figure 2C, D**). The T-distributed stochastic neighbor embedding (t-SNE) algorithm was used for selecting the first 20 principal components for the dimension reduction. 19 cell clusters were identified by Seurat (**Figure 2E**). Cluster 17 was identified as a B cell according to the differentially expressed genes and B cell-specific marker gene MS4A1(CD20) in each cluster (**Figure 2F, G**). Finally, 261 B cell marker genes were obtained.

### Construction of the risk‐scoring model

**Figure 3:**
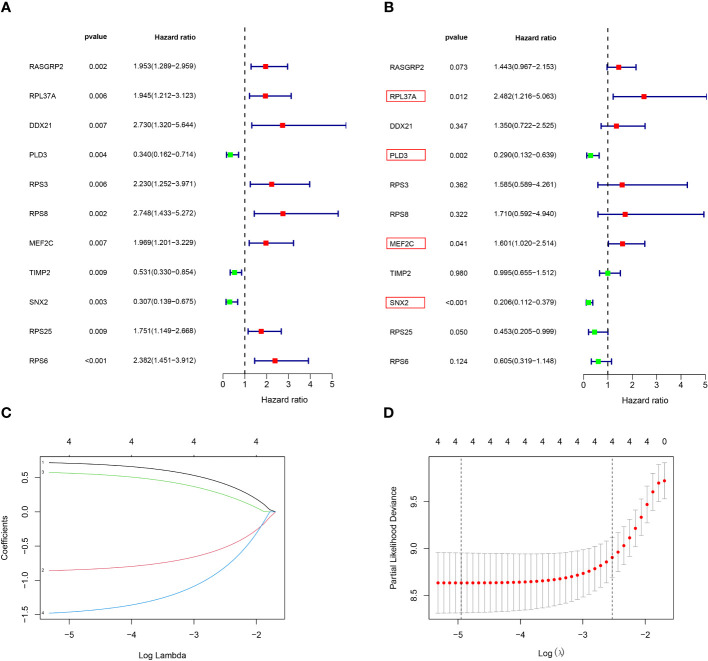
Construction of a risk‐scoring model for patients with osteosarcoma based on the TCGA database. **(A, B)** Univariate and multivariate Cox analysis demonstrated the correlation between B cell marker genes and prognosis. **(C, D)** LASSO regression analysis further screened the prognostic genes of B cell markers. (1: RPL37A, coef:0. 0.662; 2:PLD3, coef: -0.798; 3:MEF2C, coef: 0.510; 4:SNX2, Coef:-1.363).

**Figure 4:**
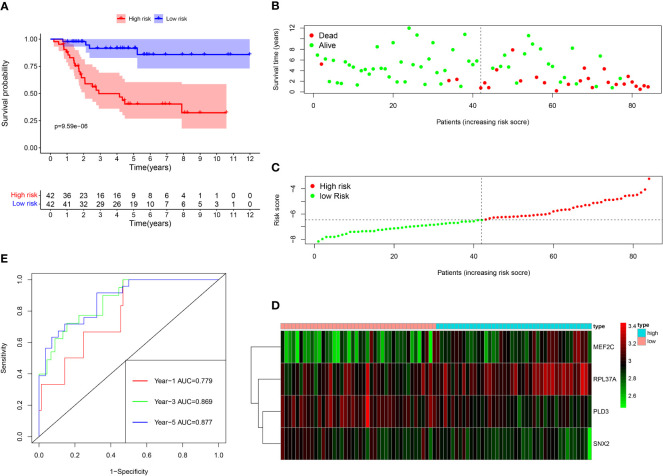
Survival analysis results of a risk‐scoring model for TCGA osteosarcoma patients. **(A)** Kaplan–Meier survival analysis of TCGA osteosarcoma patients in high‐risk and low‐risk groups. **(B) **The overall survival rate and the survival status of TCGA osteosarcoma patients. **(C)** The distribution of risk scores for each patient. **(D)** The expression levels of these 4 B cell marker genes in the high‐risk and low‐risk groups. The green color represents low expression, while the red color represents high expression. **(E)** ROC curve analysis of the risk-scoring model’s 1-year, 3‐year, and 5‐year OS. OS, overall survival, ROC, receiver operating characteristic.

**Figure 5:**
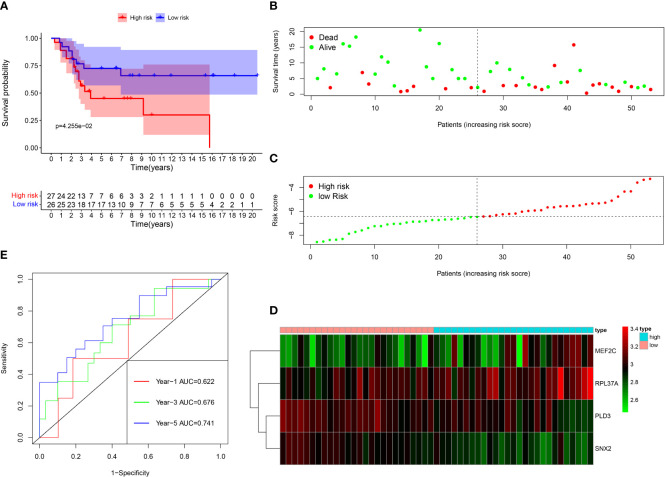
Survival analysis results of a risk‐scoring model for GEO osteosarcoma patients. **(A)** Kaplan–Meier survival analysis of GEO osteosarcoma patients in high‐risk and low‐risk groups. **(B) **The overall survival rate and the survival status of GEO osteosarcoma patients. **(C)** The distribution of risk scores for each patient. **(D)** The expression levels of these 4 B cell marker genes in the high‐risk and low‐risk groups. The green color represents low expression, while the red color represents high expression. **(E)** ROC curve analysis of the risk-scoring model’s 1-year, 3‐year, and 5‐year OS. OS, overall survival, ROC, receiver operating characteristic.

Among the 261 B cell marker genes, five of them were screened based on univariate Cox and multivariate Cox analysis (**Figure 3A, B**). LASSO regression analysis was used to identify four genes (RPL37A, MEF2C, PLD3, SNX2) and the risk score of each patient was computed (**Figure 3C, D**). The correlation of these four gene expressions with B cells was verified in the Human Protein Atlas (HPA) database (**Figure S1**). Based on the median risk score of TCGA patients, the patients were divided into the low-risk and high-risk groups. Kaplan-Meier survival analysis revealed a significantly lower time of overall survival in the high-risk than that in the low-risk group (**Figure 4A**). The survival and risk score of each osteosarcoma patient has been shown in **Figure 4B, C**. The gene heat plot shows the expression level of four genes in each patient (**Figure 4D**). Finally, the accuracy of the risk score model was estimated by the time ROC curve, and the results displayed that the AUC (area under the curve) values of patients at 1, 3, and 5 years were 0.779, 0.869, and 0.877, respectively (**Figure 4E**). In the GEO validation group, similar results were obtained. The overall survival time of osteosarcoma patients in the low-risk group was longer than that of osteosarcoma patients in the high-risk group (**Figure 5A–D**). The AUC values of 1, 3 and 5 years ROC curves of GEO patients were 0.622, 0.676, and 0.741, respectively (**Figure 5E**). In addition, PCA analysis revealed that the expression levels of the four prognostic genes significantly differentiated patients in the low- and high-risk groups in both the training and validation groups (**Figure S2**), thereby illustrating the prognostic accuracy of the developed risk-scoring model.

### Immune infiltration between the high-risk group and low-risk group

**Figure 6:**
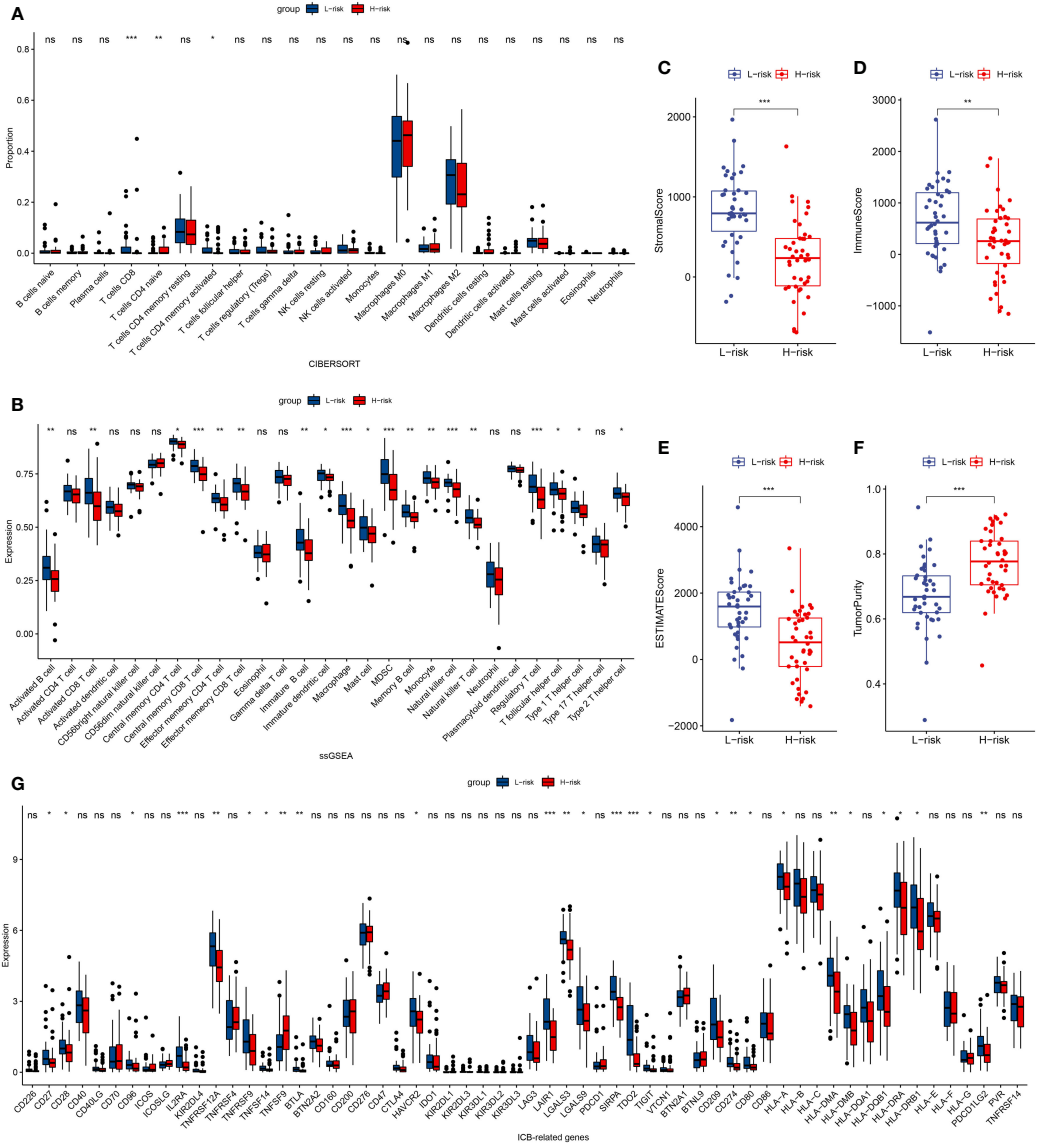
TCGA tumor microenvironment between the high-risk and low-risk groups. **(A)** Boxplots depicting the CIBERSORT scores of 22 immune cells of the high-risk patients compared to low-risk patients. **(B)** Boxplots depicting the 29 immune signature ssGSEA scores of the high-risk patients compared to low-risk patients. **(C–F)** Comparisons between the 2 groups in terms of stomal score, immune score, estimate score, and tumor purity. **(G)** Expression of ICB-related genes in high- and low-risk groups. *P < 0.05, **P < 0.01, ***P < 0.001, ns, P > 0.05.

**Figure 7:**
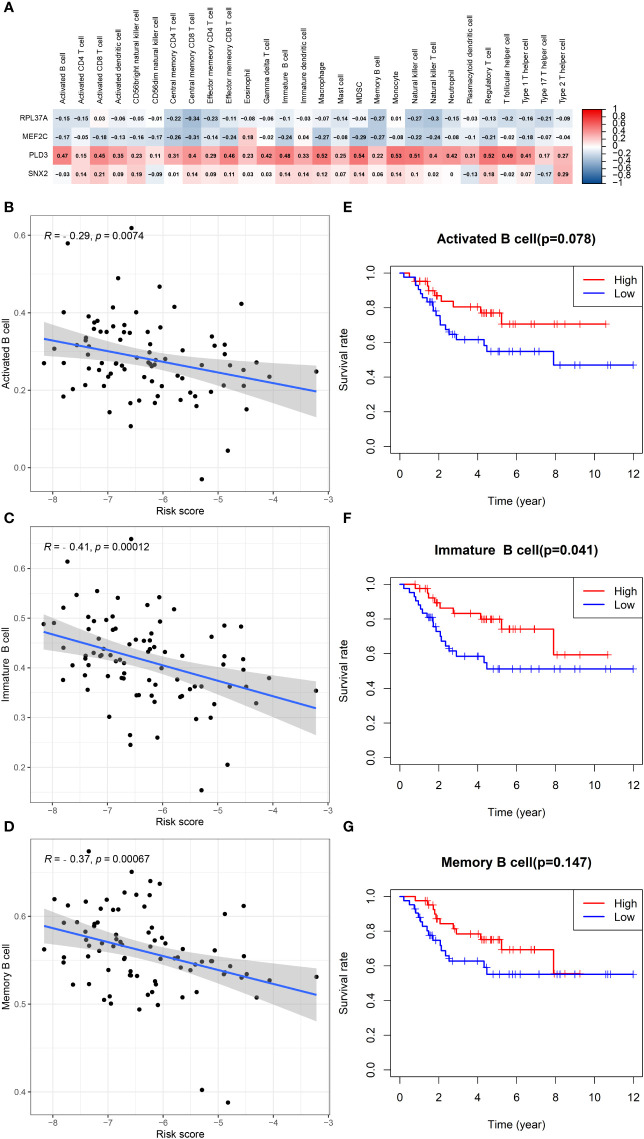
B cells in TCGA data in relation to prognosis. **(A)** Heatmap depicting the correlation between 4-gene mRNA expressions with the 22 immune cells. **(B–D)** Correlation fitted curves of risk scores with activated B cells, immature B cells, and memory B cells. **(E–G)** Survival curves grouped by median content of activated B cells, immature B cells and memory B cells, respectively.

In patients with TCGA osteosarcoma, CIBERSORT analysis showed that the low-risk group had a higher proportion of T-cells CD8 and T-cells CD4 memory activated, while a lower proportion of T-cells CD4 naive when compared with the high-risk group (**Figure 6A**). ssGSEA analysis revealed that the low-risk group had a higher content of activated B cell, activated CD8 T cell, central memory CD4 T cell, central memory CD8 T cell, effector memory CD4 T cell, effector memory CD8 T cell, immature B cell, immature dendritic cell, macrophage, mast cell, MDSC, memory B cell, monocyte, natural killer cell, natural killer T cell, regulatory T cell, T follicular helper cell, type 1 T helper cell, and type 2 T helper cell in comparison to the high-risk group (**Figure 6B**). The low-risk group also had a higher stromal score, immune score, estimates score, and lower tumor purity (**Figure 6C–F**). Lower expression of genes associated with immune checkpoint blockade in the high-risk group suggested that the low-risk group is more suitable for immune checkpoint inhibitor therapy (**Figure 6G**). It was also found that the prognostic risk genes RPL37A and MEF2C were negatively correlated with immune cells, unlike the prognostic protective genes PLD3 and SNX2 (**Figure 7A**). The survival curves validated the better probability of survival in patients with high B-cell content (**Figure 7E–G**). Moreover, the risk score obtained by B-cell marker genes in this study was inversely proportional to the B-cell content (**Figure 7B–D**). In patients with GSE21257, CIBERSORT analysis revealed that the low-risk group had a lower proportion of B cells naïve compared with the high-risk group (**Figure S3A**). ssGSEA analysis showed that the low-risk group had a higher content of activated B cell, activated CD4 T cell, activated CD8 T cell, activated dendritic cell, central memory CD8 T cell, effector memory CD8 T cell, eosinophil, immature B cell, immature dendritic cell, macrophage, mast cell, MDSC, memory B cell, natural killer cell, natural killer T cell, neutrophil, regulatory T cell, T follicular helper cell, and type 1 T helper cell (**Figure S3B**). The results of the GEO verification group were found to be roughly consistent with those of TCGA (**Figure S3**, **S4**).

### Nomogram of the prediction model

**Figure 8:**
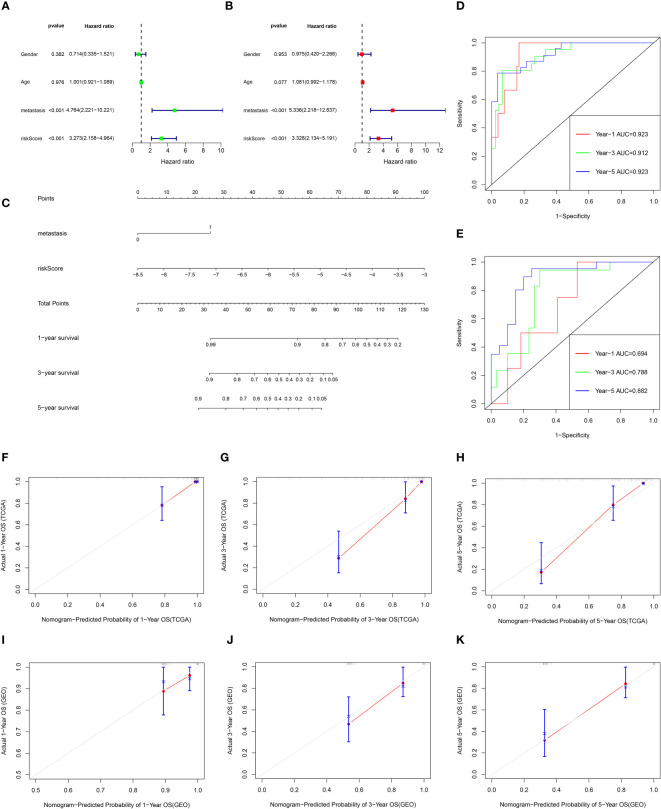
The prognostic value of clinical features and risk scores, and construct the nomogram. **(A, B)** Results of the univariate and multivariate Cox regression analyses regarding OS in the TCGA cohort. **(C)** Nomogram plots of TCGA cohort. **(D)** The ROC curve analysis of the TCGA cohort showed that AUCs of 1-year, 3‐year, and 5‐year OS was 0.923, 0.912, and 0.923, respectively. **(E)** The ROC curve analysis of the GEO cohort showed that AUCs of 1-year,3‐year, and 5‐year OS was 0.694, 0.788, and 0.882, respectively. **(F–H)** Calibration curves for 1, 3, and 5-year OS of TCGA data. **(I–K)** Calibration curves for 1, 3, and 5-year OS of GEO data.

Univariate Cox and multivariate Cox analysis revealed that the risk score and metastasis were important prognostic factors for osteosarcoma patients in the TCGA database (**Figure 8A, B**). Based on these two factors, a nomogram was set up, the TCGA data were internally verified and the GSE21267 data was externally verified (**Figure 8C**). In the validation of the time ROC curve of TCGA data, the AUC values of 1, 3, and 5 years were 0.923, 0.912, and 0.923, respectively (**Figure 8D**). In the verification of the time ROC curve of geo data, the AUC values of 1, 3, and 5 years were 0.694, 0.788, and 0.882, respectively (**Figure 8E**). The C-index calibration plots for both the training and validation groups reveal the predictive accuracy of the prognostic model (**Figure 8F–K**).

### Gene features set enrichment analysis

**Figure 9:**
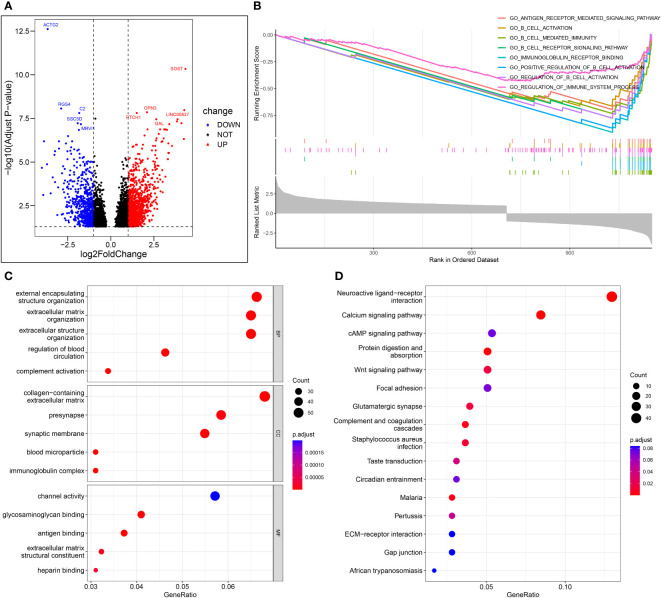
Enrichment analysis. **(A)** Volcano map of differential genes in the high-risk and low-risk groups. **(B)** The important pathway of GSEA analysis. **(C)** The most significant and shared KEGG analysis in the TCGA. **(D)** The most significant and shared GO analysis in the TCGA.

**Figure 10:**
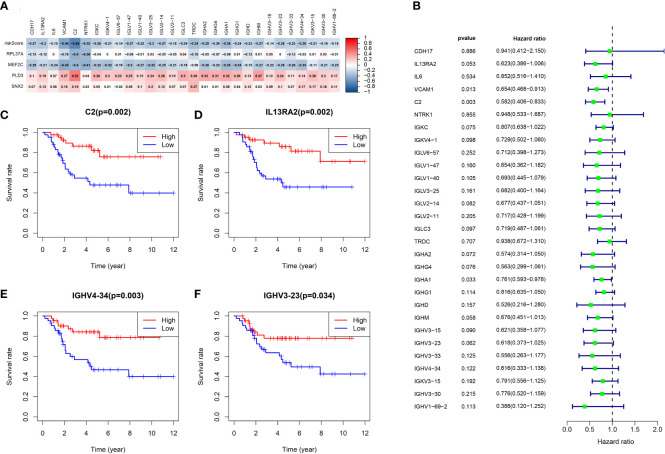
Analysis of B-cell activation pathway genes. **(A)** Heat map of risk score and correlation analysis of four prognostic genes with 29 B-cell activation pathway genes. **(B)** Univariate cox analysis of 29 genes and overall survival. **(C-F)** Survival curves of four genes associated with B-cell activation.

**Figure 9A** shows the volcano map of differential genes in the high-risk and low-risk groups. GSEA enrichment analysis displayed that the differential genes were related to B cell function, antigen-antibody binding, and immunomodulatory pathway (**Figure 9B**). GO enrichment analysis showed the higher involvement of the differential genes in the biological process of external encapsulating structure organization (**Figure 9C**). KEGG enrichment analysis displayed the relation between the differential memory to the Neuroactive ligand-receptor interaction, cAMP signaling pathway, Wnt signaling pathway, and other pathways (**Figure 9D**). A total of 29 genes were included in the five pathways associated with B-cell activation analyzed by GSEA enrichment. The 2 prognostic risk genes and risk scores were negatively correlated with these 29 genes, while the 2 prognostic protective genes were positively correlated with these 29 genes (**Figure 10A**). Univariate cox analysis of these 29 genes showed them to be prognostic protective genes for osteosarcoma (although many genes had P > 0.05), which also demonstrates that B-cell activation drives better outcomes in patients with osteosarcoma (**Figure 10B**). The survival curves were plotted distinguishing between high and low groups of patients by median gene expression (osteosarcoma patients with TCGA), showing four of these genes with p-values < 0.05, all of whom are highly expressed and represent a better prognosis (**Figure 10C–F**). These represent the association of these prognostic genes with B cells and a good predictive ability of the risk score model.

### Content of B cells in the immune microenvironment of osteosarcoma

**Figure 11:**
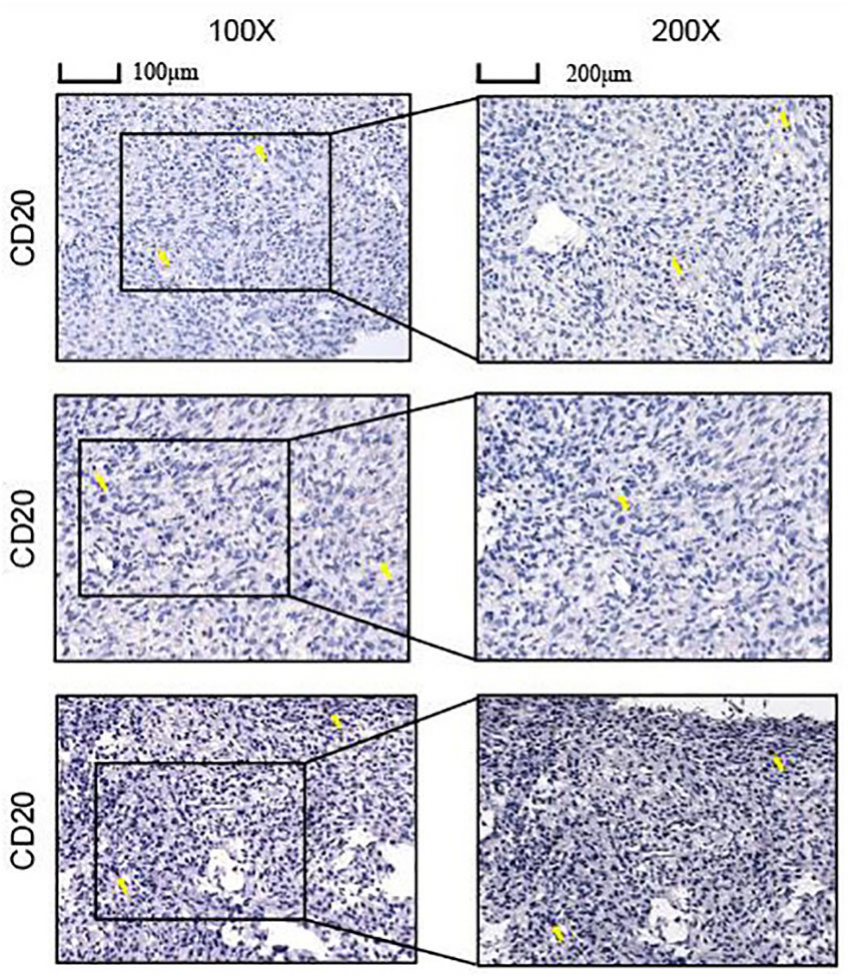
Immunohistochemistry (IHC) staining of OS using anti-CD20 antibody staining.

Immunohistochemical staining revealed B cells to be an important component of the immune infiltrate in osteosarcoma, suggesting its significant influence on the development of osteosarcoma (**Figure 11**).

## Discussion

In this study, a prognostic model consisting of four B cell marker genes, RPL37A, MEF2C, PLD3, and SNX2 was constructed. RPL37A and MEF2C were found to be associated with the risk of prognosis, while PLD3 and SNX2 were found to protect the prognosis. RPL37A is located in the cytoplasm and encodes ribosomal proteins, belonging to the L37AE family of ribosomal proteins. RPL37A has been reported as a prognostic risk gene for osteosarcoma, which is consistent with the results of this study (10). MEF2C is necessary for the normal development of platelets and megakaryocytes and the production of bone marrow B-lymphocytes. It also plays a significant role in the survival and proliferation of B cells in response to BCR (B-cell receptor) stimulation, effective IgG1 antibody response to T cell-dependent antigens, and the normal induction of germinal center B cells. The latest investigation by Tan et al. reported an indirect correlation between MEF2C and the adverse prognosis of osteosarcoma (11). The results of this investigation further confirm this correlation. It is interesting to observe that MEF2C, a significant gene affecting the development of B cells, leads to a poor prognosis of osteosarcoma. PLD3 can regulate inflammatory cytokine response through the degradation of nucleic acid. PLD3 has also been shown to be a prognostic protective gene for osteosarcoma, but this is the first time we have confirmed that it is also a B-cell marker gene for osteosarcoma (12–14). SNX2 belongs to the sorting nexin family and plays a role in the transport of intracellular cargo proteins. At present, no investigations are reporting the role of SNX2 in osteosarcoma. To the best of our knowledge, this is the first investigation reporting SNX2 as a prognostic protective gene for osteosarcoma and the significant association of B cells with the prognosis of osteosarcoma patients. This investigation also established a prognostic model based on B cell marker genes.

The tumor immune infiltration plays an indispensable role in the invasion and progression of cancer (5, 6). The effect of B cells on tumors is a complex process and has not been studied until now. B cells can suppress anti-tumor T cells by secreting IL-10 and promote tumorigenesis by secreting antibodies exacerbating chronic inflammation (15). Experiments on mice models revealed that the presence of anti-IgM antibodies and the depletion of B-cells reduced the incidence of metastatic breast cancer compared to normal mice (16). Another study reported delayed growth of colon cancer, thymoma, and melanoma in defective B-cell mice (17).

Most of these investigations suggested that B cells promote tumor progression, and due to the absence of spontaneous anti-tumor cell antibodies, much of the research was majorly focused on T cells (especially CD8^+^ T cells), which were thought to be the primary anti-tumor immune cells. However, recent investigations have displayed the close association of B cells to the prognosis of tumor patients and the response to immunotherapy (15, 18). To the contrary, DiLillo et al. reported that treatment of tumor-bearing mice with anti-CD20 antibody resulted in increased size of melanoma and increased number of lung metastases, and concluded that B-cell depletion impairs IFN-Y-producing T helper cells (19). B cells are usually organized into tertiary lymphoid structures, which are immune cell immunocyte aggregates with lymphoid node-like characteristics (20, 21). TLS is an ectopic lymphoid organ that develops when tissue is chronically stimulated by antigens, and Lu et al. has reported that chemotherapy can increase immune inflammation and also TLS (22). The presence and amount of TLS are associated with favorable prognoses in several cancers (23). Most of the PD-1 and PD-L1 in some tumors are located in TLS, and the frequent contact between PD-1 and PD-L1 or macrophage cells indicates that they are immune checkpoint suppression (ICI) (24, 25). Immune checkpoint inhibitors have revolutionized the treatment of cancer with their aim to revitalize depleted T cells and may be more effective in highly mutated tumors such as melanoma and non-small cell lung cancer, leading to the over-expression of tumor-specific antigens. However, many patients are insensitive to immune checkpoint suppression therapies, necessitating the need to investigate other immune cells in the tumor immune microenvironment (15). B cells in tertiary lymphoid structures form germinal centers, actively secrete antibodies, recognize tumor-associated antigens, and promote anti-tumor immunity of T cells by providing them with tetravalent signals, such as CD80 and CD86 (18, 20, 21). In some cases, B cells also recognize extracellular domains, thereby redirecting the cytotoxicity of natural killer (NK) and bone marrow cells to tumor cells (24). B cells can also activate macrophages through antibody-dependent phagocytosis, where IgG antibodies can also be directly involved in anti-tumor (23, 26). This is consistent with the results of this investigation in which risk scores were negatively correlated with NK cells and helper T cells. By analyzing the relationship between immune-related genes and prognosis in soft tissue sarcoma, Petitprez et al. reported a positive correlation between B-cell infiltration and tertiary lymphoid structures in soft tissue sarcoma and the prognosis of patients and their effect on immunotherapy (27). A positive correlation between B cell infiltration and patients’ immunotherapy outcomes, with an important role in maintaining T cell function, has also been reported (28, 29). Tumor antigens were also found to enhance the anti-tumor effects of CD8 T cells by interacting with B cells and CD4 T cells (18). In this study, the risk score was inversely correlated with both CD4 T cells and CD 8 T cells. A cohort study including 25 cancers reported that B cells were prognostically preferable in 50% of tumors, detrimental in 9%, and not associated with prognosis in 41% of tumors (30). It was found in this study that the immune infiltration in the low-risk group was notably different from that in the high-risk group, which may be due to the difference between B cells further affecting natural killer cells and T cells. The expression of immune checkpoint receptors was also remarkably different between the low-risk and high-risk groups. The results suggest that the developed predicted model can distinguish the immune infiltration of osteosarcoma patients and provide guidance for immunotherapy for patients with osteosarcoma.

For further verification, the differential genes between the low-risk and the high-risk groups were enriched and analyzed. Through GSEA enrichment analysis, it was found that it was mainly enriched in B cell-related pathways. The genes associated with B-cell activation were broadly those of the immunoglobulin and complement systems. These genes are also prognostic protective genes for osteosarcoma. These results further confirm the present study. Interestingly, the results of the KEGG enrichment analysis include Wnt signaling pathway that plays an indispensable role in the proliferation and metastasis of osteosarcoma (31, 32). Through this study, it can be speculated that the Wnt signal pathway may participate in the regulation of tumor immune infiltration. Although this requires further validation, it provides a guiding direction for the study of immune infiltration of osteosarcoma.

## Conclusion

B cell marker gene was obtained based on single-cell sequencing and a prognostic model composed of four genes was constructed. This model demonstrated good predictive performance and provides a standard for predicting survival in patients with osteosarcoma. The prognostic model developed in this study also distinguishes between patients with good and poor immune infiltration, and a significant difference in the expression of immune checkpoint receptors in the low-risk group and high-risk group was observed. The results obtained in this investigation can therefore guide the immunotherapy of patients with osteosarcoma and improve the rate of their survival.

## Data availability statement

The datasets presented in this study can be found in online repositories, the names of the repositories/accession numbers can be found in the article/**Supplementary Material**.

## Author contributions

ZZ, JZ, XL: Conceptualization, Methodology, Software, Investigation, Formal. Analysis, Writing - Original Draft. JP, GW and BS: Conceptualization, Funding Acquisition, Resources, Supervision, Writing - Review & Editing. All authors contributed to the article and approved the submitted version.

## Funding

This work was supported by clinical project of Shanghai Municipal Health Commission (201940283).

## Conflict of interest

The authors declare that the research was conducted in the absence of any commercial or financial relationships that could be construed as a potential conflict of interest.

## Publisher’s note

All claims expressed in this article are solely those of the authors and do not necessarily represent those of their affiliated organizations, or those of the publisher, the editors and the reviewers. Any product that may be evaluated in this article, or claim that may be made by its manufacturer, is not guaranteed or endorsed by the publisher.

## Supplementary material

Supplementary Figure S1 Immune cell expression of four B-cell marker genes. **(A)** RPL37A. **(B)** MEF2C. **(C)** PLD3. **(D)** SNX2.

Supplementary Figure S2 Principal component analysis. **(A)** TCGA. **(B)** GSE21257.

Supplementary Figure S3 GSE21257 tumor microenvironment between the high-risk and low-risk groups. **(A)** Boxplots depicting the CIBERSORT scores of 22 immune cells of the high-risk patients compared to low-risk patients. **(B)** Boxplots depicting the 29 immune signature ssGSEA scores of the high-risk patients compared to low-risk patients. **(C–F)** Comparisons between the 2 groups in terms of stomal score, immune score, estimate score, and tumor purity. **(G)** Expression of ICB-related genes in high- and low-risk groups.

Supplementary Figure S4 B cells in GSE21257 data in relation to prognosis. **(A)** Heatmap depicting the correlation between 4-gene mRNA expressions with the 22 immune cells. **(B–D)** Correlation fitted curves of risk scores with activated B cells, immature B cells, and memory B cells. **(E–G)** Survival curves grouped by median content of activated B cells, immature B cells and memory B cells, respectively.
