# Supplementary figures and images for: Identification of B cell marker genes based on single-cell sequencing to establish a prognostic model and identify immune infiltration in osteosarcoma
Source: Front Immunol. 2022 Dec 7;13:1026701. doi: 10.3389/fimmu.2022.1026701 (PMC9774034; doi:10.3389/fimmu.2022.1026701)

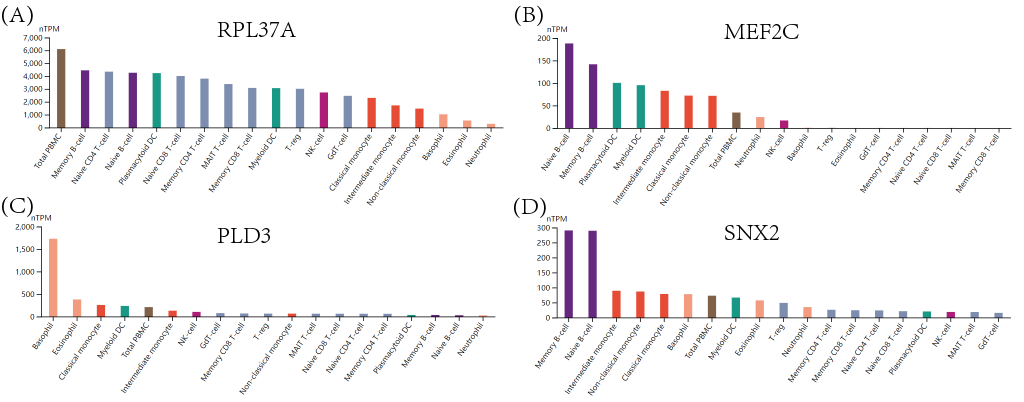

Supplement: Supplementary Figure S1 — Immune cell expression of four B-cell marker genes. (A) RPL37A. (B) MEF2C. (C) PLD3. (D) SNX2. [file Image_1.tif]

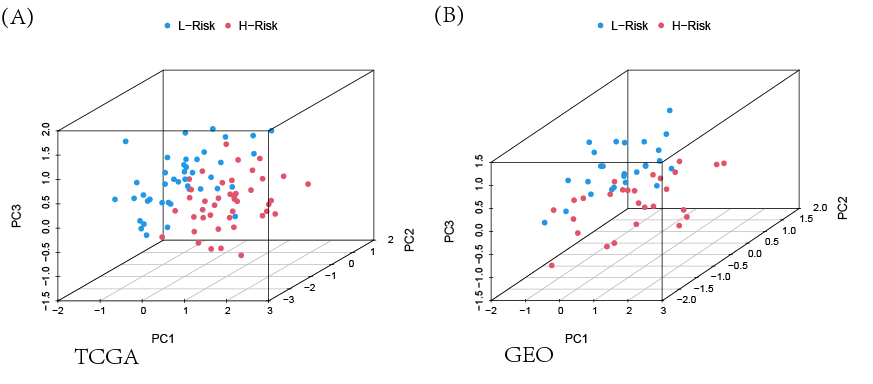

Supplement: Supplementary Figure S2 — Principal component analysis. (A) TCGA. (B) GSE21257. [file Image_2.tif]

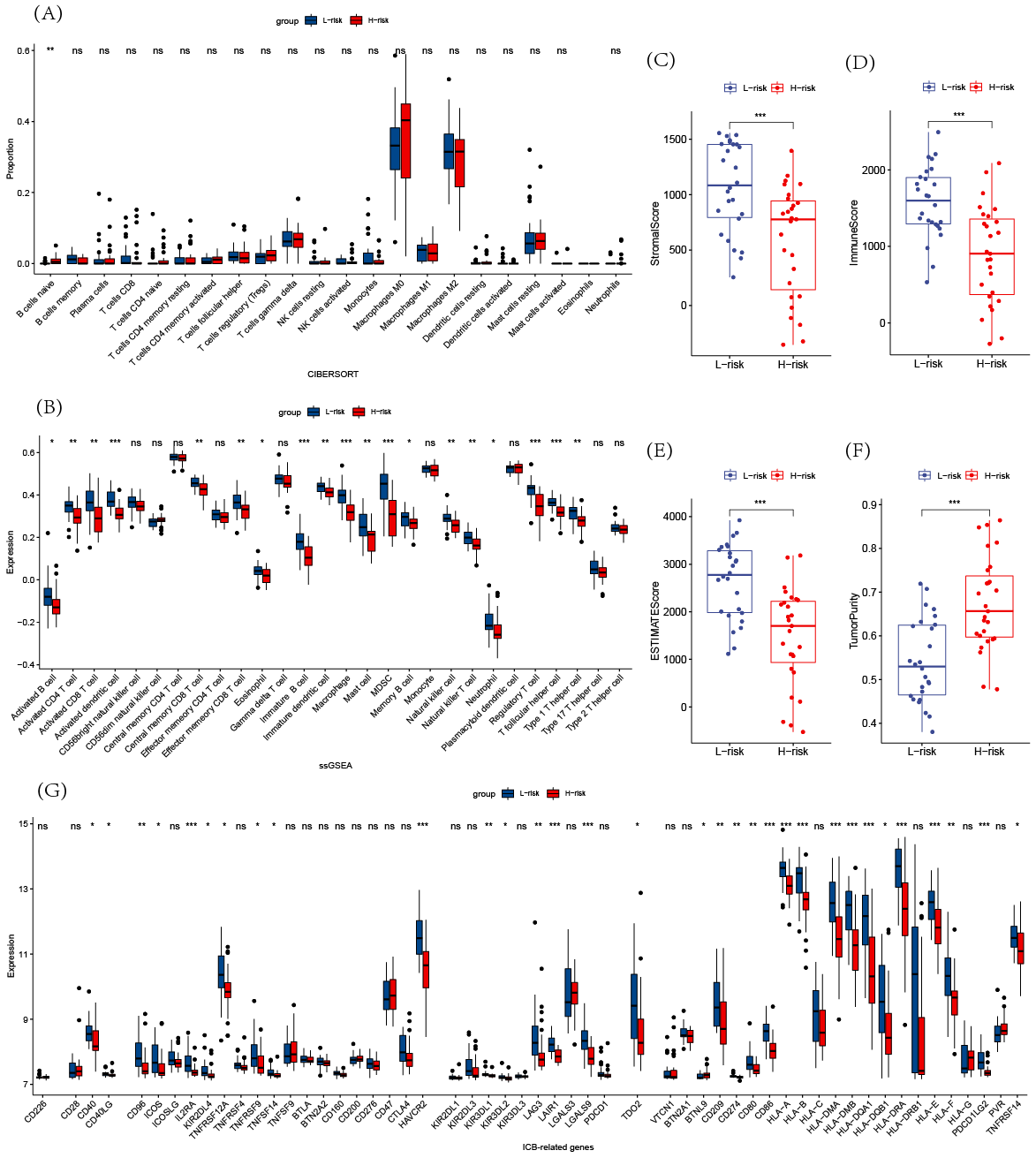

Supplement: Supplementary Figure S3 — GSE21257 tumor microenvironment between the high-risk and low-risk groups. (A) Boxplots depicting the CIBERSORT scores of 22 immune cells of the high-risk patients compared to low-risk patients. (B) Boxplots depicting the 29 immune signature ssGSEA scores of the high-risk patients compared to low-risk patients. (C–F) Comparisons between the 2 groups in terms of stomal score, immune score, estimate score, and tumor purity. (G) Expression of ICB-related genes in high- and low-risk groups. [file Image_3.tif]

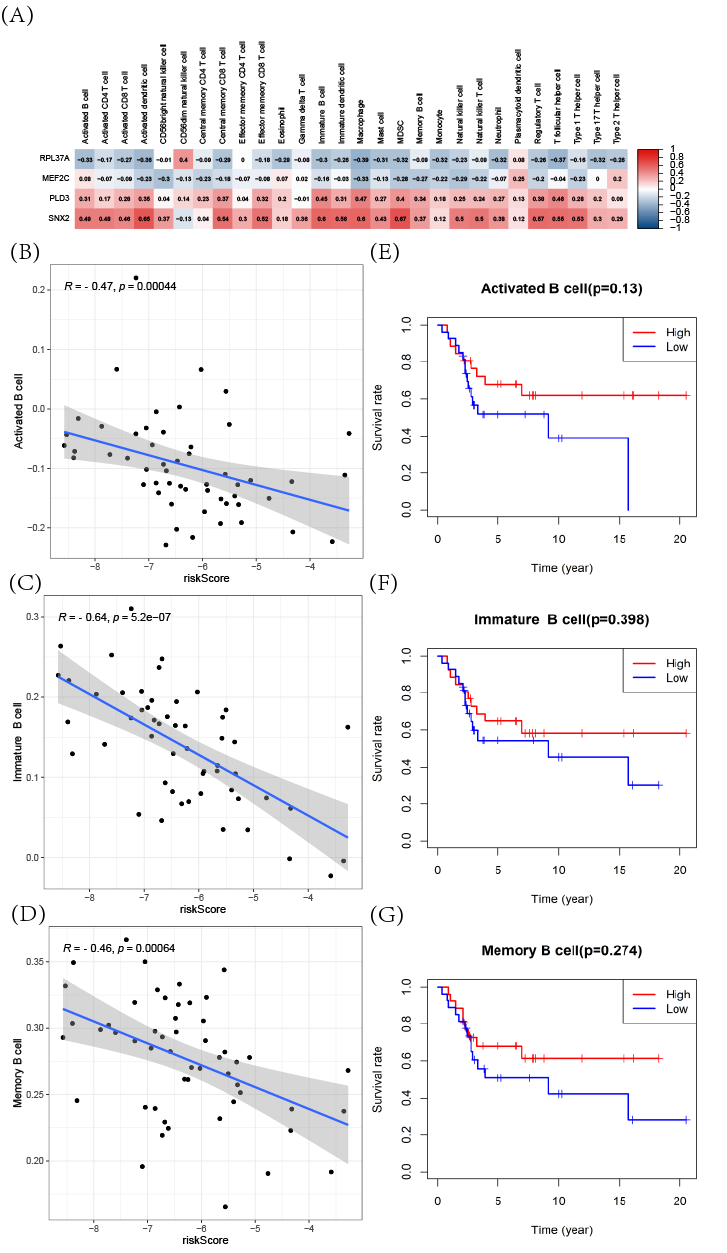

Supplement: Supplementary Figure S4 — B cells in GSE21257 data in relation to prognosis. (A) Heatmap depicting the correlation between 4-gene mRNA expressions with the 22 immune cells. (B–D) Correlation fitted curves of risk scores with activated B cells, immature B cells, and memory B cells. (E–G) Survival curves grouped by median content of activated B cells, immature B cells and memory B cells, respectively. [file Image_4.tif]
