# Supplementary material for: Identification of B cell marker genes based on single-cell sequencing to establish a prognostic model and identify immune infiltration in osteosarcoma
Source: Front Immunol. 2022 Dec 7;13:1026701. doi: 10.3389/fimmu.2022.1026701 (PMC9774034; doi:10.3389/fimmu.2022.1026701)
Supplement: Supplementary file 5 [file Table_1.docx]

TABLE S1 Baseline patient data in training and validation setsr

| ITEM | Training cohort (TCGA n = 84) | Validation cohort (GSE21257 n = 53) |
| --- | --- | --- |
| Age, n(%)  <18  ≥18 | 66(78.6%)  18(21.4%) | 34(64.2%)  19(35.8%) |
| Gender, n(%)  Female  Male | 37(44.0%)  47(56.0%) | 19(35.8%)  34(64.2%) |
| Metastasis, n(%)  Nonmetastatic  Metastasis | 63(75.0%)  21(35.0%) | 19(35.8%)  34(64.2%) |
| Status, n(%)  Alive  Dead | 57(67.9%)  27(32.1%) | 30(62.3%)  23(37.7%) |

Abbreviation: TCGA, The Cancer Genome Atlas.
